# Kahweol decreases hepatic fibrosis by inhibiting the expression of connective tissue growth factor via the transforming growth factor-beta signaling pathway

**DOI:** 10.18632/oncotarget.19756

**Published:** 2017-08-01

**Authors:** Hye-Young Seo, Yun-A Jung, So-Hee Lee, Jae Seok Hwang, Keun-Gyu Park, Mi-Kyung Kim, Byoung Kuk Jang

**Affiliations:** ^1^ Department of Internal Medicine, Keimyung University School of Medicine, Daegu, South Korea; ^2^ Institute for Medical Science, Keimyung University School of Medicine, Daegu, South Korea; ^3^ Department of Internal Medicine, Kyungpook National University School of Medicine, Daegu, South Korea

**Keywords:** kahweol, hepatic fibrosis, CTGF, TGF-β

## Abstract

Kahweol is a diterpene molecule found in *Coffea Arabica* beans. Previous studies have shown that coffee reduces liver fibrosis, but it is not clear which component of coffee has the protective effect. In this study, we examined whether kahweol has a protective effect on hepatic fibrosis *in vivo* and *in vitro*. Kahweol decreased hepatic fibrosis by inhibiting connective tissue growth factor (CTGF) expression in thioacetamide (TAA)-treated mice. The expression of phospho-Smad3, signal transducer and activator of transcription 3 (STAT3), extracellular signal-regulated kinases (ERK), and c-Jun N-terminal protein kinase (JNK) increased in the livers of TAA-treated mice and decreased in the kahweol-treated group. Kahweol significantly decreased the expression of transforming growth factor beta (TGF-β)-stimulated type I collagen and CTGF expression *in vitro*. In addition, kahweol significantly decreased the expression of Smad3, STAT3, ERK and JNK, which are involved in the induction of CTGF expression by TGF-β in hepatocytes, but not in HSCs. These results suggest that kahweol may be a new candidate for treatment of liver fibrosis.

## INTRODUCTION

Coffee is the most widely consumed, pharmacologically active beverage in the world. It is a complex mixture of more than 1000 compounds such as caffeine, chlorogenic acid, melanoidins, and diterpenes [1–3]. The various antioxidants in coffee have protective effect against tumors, diabetes, and Parkinson's disease [1, 4, 5]. Kahweol, a diterpene molecule, is one of the constituents of coffee from the beans of *Coffea arabica*. This compound is present in unfiltered coffee drinks such as espresso, boiled coffee, or Greek coffee [6]. It is also well known that kahweol is anti-inflammatory and anti-angiogenic, and is therefore anti-cancerous [7, 8].

Hepatic fibrosis is characterized by excessive and abnormal deposition of extracellular matrix (ECM) proteins. Hepatic stellate cells (HSCs) are activated during the progression of liver fibrosis and are a major source of ECM proteins in the liver. Following injury to the liver, quiescent HSCs are transformed into myofibroblast like cells, which are characterized by the expression of alpha smooth muscle actin (α-SMA) and deposition of collagens [9–11].

Connective tissue growth factor (CTGF/CCN2) is a secreted matricellular protein with very complex biology. It participates in the regulation of biological processes, including cell adhesion, migration, proliferation and angiogenesis, and extracellular matrix production [12]. CTGF is expressed in response to tissue injury and is used as a biomarker of hepatic fibrosis [13]. Earlier studies reported that transforming growth factor beta (TGF-β) induced CTGF expression in hepatocytes and this CTGF is associated with the development of hepatic fibrosis [14, 15]. TGF-β signaling and its signaling pathway members, Smads, are regarded as the key fibrogenic signals contributing to liver fibrosis. Signal transducer and activator of transcription 3 (STAT3) is involved in activating CTGF. STAT3 activation accompanies enhanced phospho-Smad3 and CTGF expression in liver fibrosis.

Previous studies have shown that coffee decreases hepatic fibrosis by decreasing the expression of CTGF [16, 17]. Although there is some evidence that coffee has protective effect against hepatic fibrosis, it is not clear which constituents of coffee have this protective effect. In this study, we investigated whether kahweol has a protective effect against hepatic fibrosis in *in vitro* and *in vivo* models.

## RESULTS

### Kahweol decreased TAA-induced hepatic fibrosis

**Figure 1:**
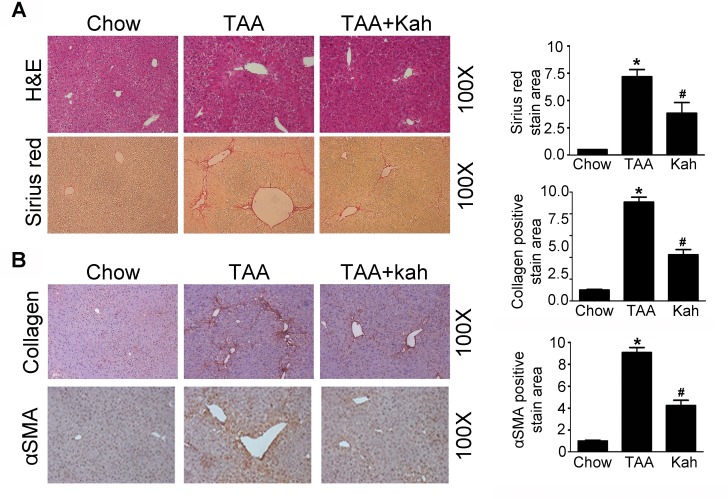
Kahweol decreases TAA-induced hepatic fibrosis in mice **(A)** Representative images of hematoxylin and eosin (H&E) staining and sirius red staining of liver tissue sections from mice after TAA-injection. Areas of positive staining with Sirius red were quantitated by computer-based morphometric analysis. Data are means ± SEM of five random fields from each liver. Magnification: 100X. ^*^P < 0.05 compared with chow group, ^#^*P* < 0.05 compared with TAA-injection group. **(B)** Representative images of immunohistochemical staining of liver tissue sections from mice after TAA-injection. Areas of positive staining with antibodies against type I collagen and α-SMA were quantitated by computer-based morphometric analysis. Data are means ± SEM of five random fields from each liver. Magnification: 100X. ^*^P < 0.05 compared with chow group, ^#^*P* < 0.05 compared with TAA-injection group.

**Figure 2:**
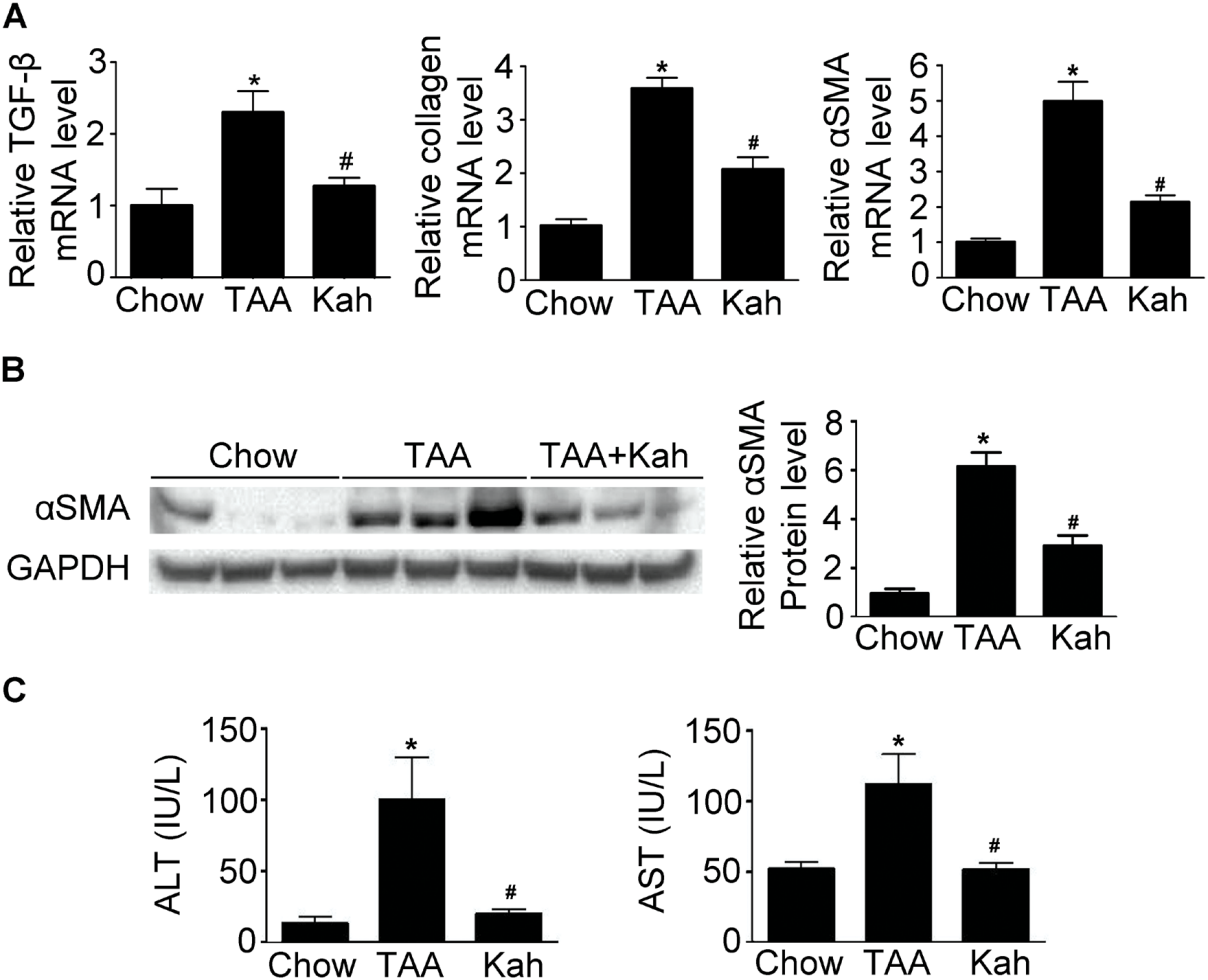
Kahweol decreases collagen and α-SMA expression and serum transaminase levels in liver **(A)** Representative real-time RT-PCR analysis of type I collagen and α-SMA mRNA expression in the liver. Data in the bar graph are mean ± SEM of three independent measurements. ^*^P < 0.05 compared with chow group, ^#^*P* < 0.05 compared with TAA-injection group. **(B)** Representative western blot analysis of α-SMA protein expression in the liver. Data represented in the bar graph are the mean ± SEM of three independent measurements. ^*^P < 0.05 compared with chow group, ^#^*P* < 0.05 compared with TAA-injection group. **(C)** Effect of kahweol on serum ALT and AST levels in TAA-treated mice. Serum ALT and AST levels were determined in serum sample by colorimetric assays. Data in the bar graph are mean ± SEM of three independent measurements. ^*^P < 0.05 compared with chow group, ^#^*P* < 0.05 compared with TAA-injection group.

To determine whether kahweol has protective effects against hepatic fibrosis, we first examined the change in hepatic pathology in a thioacetamide (TAA)-induced hepatic fibrosis mouse model. Hematoxylin and eosin (H&E) and Sirius red staining revealed markedly elevated fibrosis following TAA treatment. However, TAA-induced hepatic fibrosis was significantly decreased by kahweol treatment (Figure 1A). Furthermore, immunohistochemical (IHC) staining showed that the kahweol-treated group had significantly reduced hepatic expression levels of type I collagen and α-SMA (Figure 1B). Real-time PCR and western blot analysis also revealed that the expression of TGF-β, collagen and α-SMA were increased in the TAA-treated group, and this effect was inhibited by kahweol treatment (Figure 2A and 2B). We also examined the changes in serum alanine transaminase (ALT) and aspartate transaminase (AST) levels. The levels of ALT and AST were significantly higher in the TAA-treated group than in the normal control group. The kahweol-treated group displayed significantly lower levels of serum ALT and AST (Figure 2C). Taken together, these findings suggest that kahweol has protective effects against TAA-induced hepatic fibrosis.

### Kahweol decreased CTGF expression

**Figure 3:**
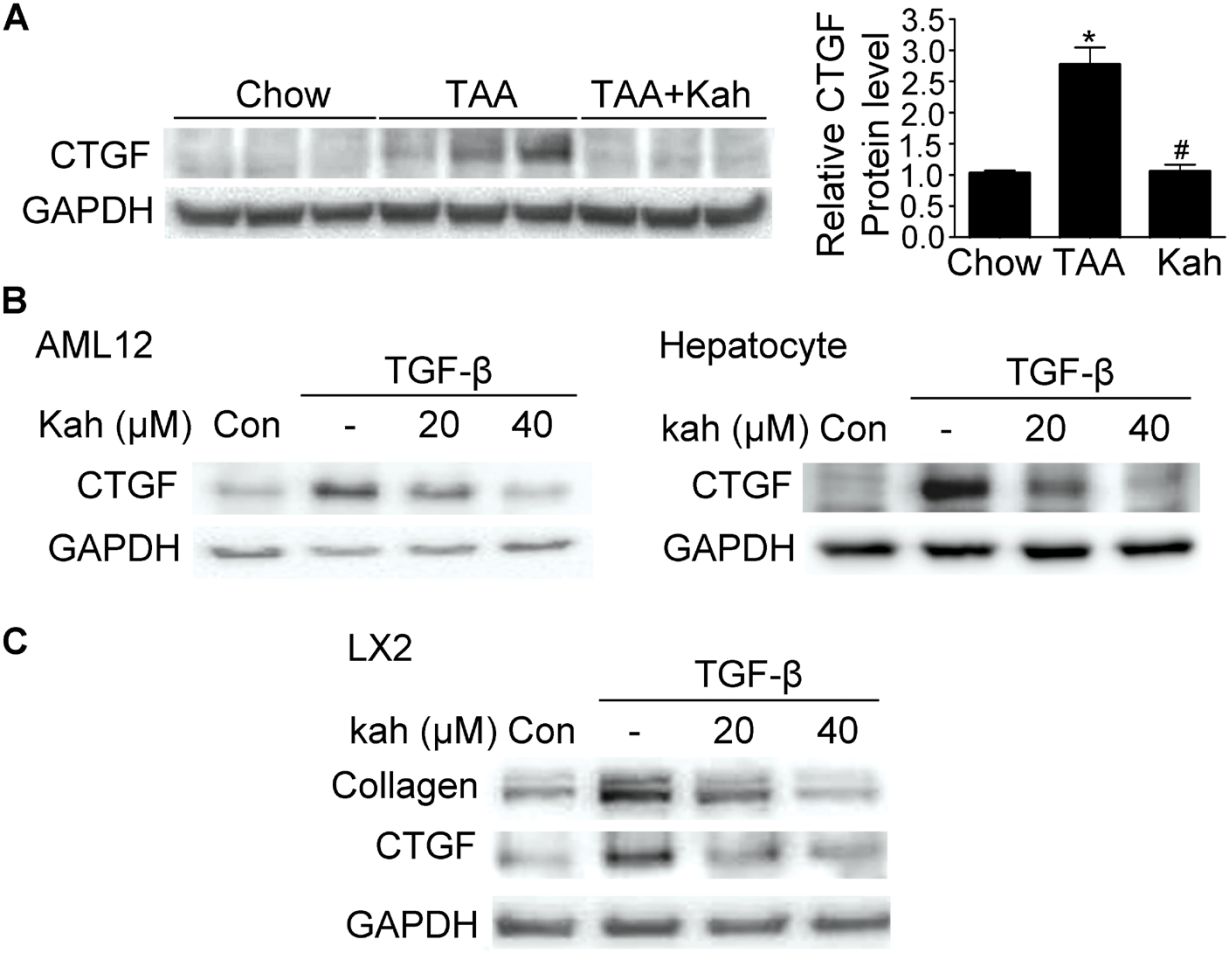
Effect of kahweol on CTGF expression **(A)** Representative western blot analysis of CTGF protein expression in the liver. Data in the bar graph are mean ± SEM of three independent measurements. ^*^P < 0.05 compared with chow group, ^#^*P* < 0.05 compared with TAA-injection group. **(B)** Western blot analysis showing the effect of kahweol on TGF-β-induced CTGF protein expression. AML12 cells and primary hepatocytes were incubated with various concentrations of kahweol with TGF-β for 24 h. **(C)** Western blot analysis showing the effect of kahweol on TGF-β-induced CTGF protein expression in LX2 cells.

CTGF is a strongly profibrogenic molecule that is overexpressed in fibrotic liver disease and mainly upregulated by TGF-β. The protein and mRNA expression of CTGF and TGF-β were upregulated in the livers of the TAA-treated group (Figure 3A). However, kahweol decreased the TAA-induced CTGF and TGF-β expression. Consistent with the results of the *in vivo* study, kahweol significantly reduced TGF-β-stimulated type I collagen expression in LX2 cells. Kahweol also decreased TGF-β-stimulated CTGF expression in AML12 cells, primary hepatocytes, and LX2 cells (Figure 3B and 3C and Supplementary Figure 1).

### Kahweol inhibited phospho-Smad3 expression

**Figure 4:**
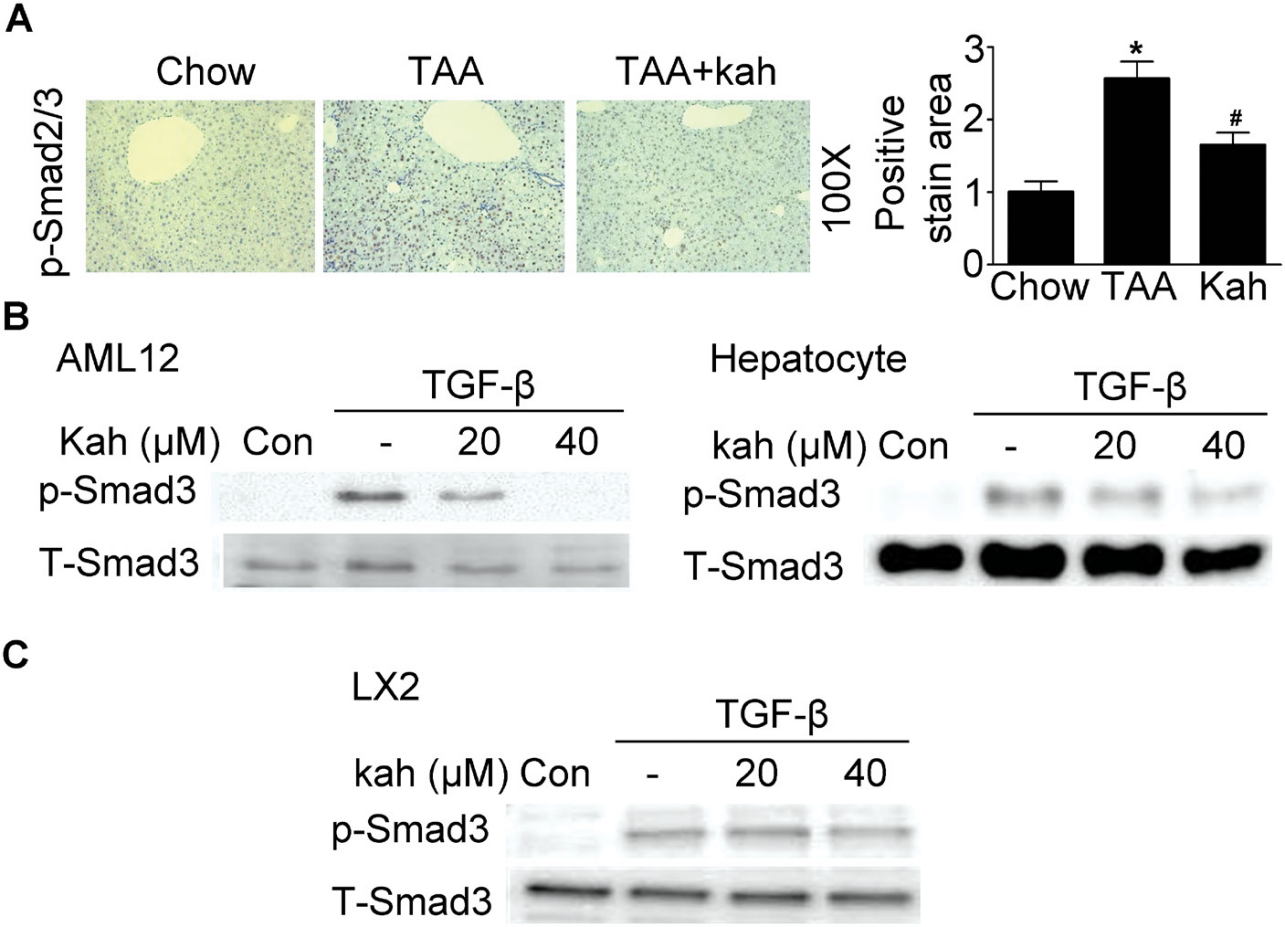
Effect of kahweol on Smad3 phosphorylation **(A)** Representative images of phospho-Smad2/3 staining of liver tissue sections from mice after TAA-injection. Areas of positive staining with phospho-Smad2/3 were quantitated by computer-based morphometric analysis. Data are mean ± SEM of five random fields from each liver. Magnification: 100X. ^*^P < 0.05 compared with chow group, ^#^*P* < 0.05 compared with TAA-injection group. **(B)** Western blot analysis showing the effect of kahweol on TGF-β-induced phospho-Smad3 expression. AML12 cells and primary hepatocytes were incubated with various concentrations of kahweol with TGF-β for 1 h. **(C)** Western blot analysis showing the effect of kahweol on TGF-β-induced phospho-Smad3 expression in LX2 cells.

The TGF-β/Smad pathway is one of the most important pathways among the multiple signaling pathways that affect CTGF expression [18]. Therefore, we investigated whether kahweol inhibits phospho-Smad3 expression. Expression of phospho-Smad3 and its subsequent nuclear translocation are critical steps in the signaling cascade that results in hepatic fibrosis. As expected, in the TAA-treated group, Smad2 and Smad3 accumulated in the nucleus (Figure 4A). However, the nuclear translocation of Smad2/3 was impaired in the kahweol-treated group. Consistent with the *in vivo* findings, kahweol also inhibited TGF-β-stimulated phospho-Smad3 protein expression in both AML12 cells and primary hepatocytes (Figure 4B). Kahweol decreased type I collagen expression, but did not decrease TGF-β-stimulated phospho-Smad3 expression in LX2 cells (Figure 4C).

### Kahweol inhibited phospho-STAT3 expression

**Figure 5:**
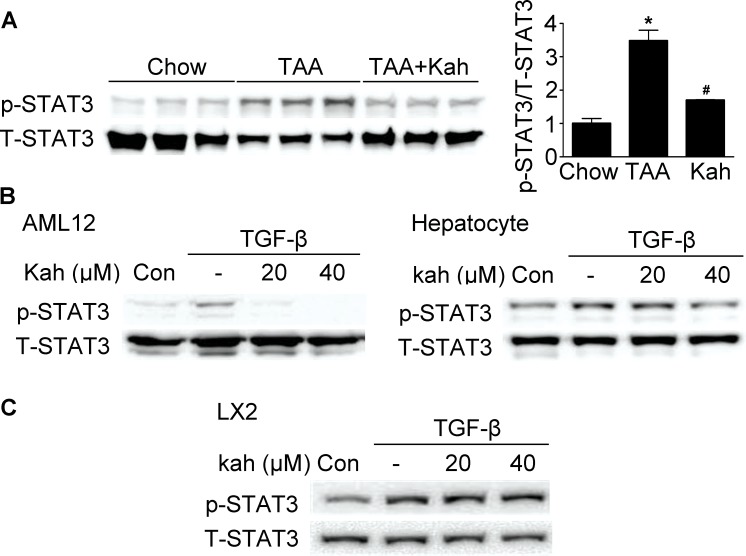
Effect of kahweol on STAT3 phosphorylation **(A)** Representative western blot analysis of phospho-STAT3 expression in the liver. Data in the bar graph are the mean ± SEM of three independent measurements. ^*^P < 0.05 compared with chow group, ^#^*P* < 0.05 compared with TAA-injection group. **(B)** Western blot analysis showing the effect of kahweol on TGF-β-induced phospho-STAT3 expression. AML12 cells and primary hepatocytes were incubated with various concentrations of kahweol with TGF-β. **(C)** Western blot analysis showing the effect of kahweol on TGF-β-induced phospho-STAT3 expression in LX2 cells.

Next, we investigated whether the inhibitory effects of kahweol on hepatic fibrosis were mediated via STAT3, because hepatic fibrosis is associated with STAT3 phosphorylation [19]. TAA treatment increased phospho-STAT3 expression in the livers of the TAA-treated group and kahweol decreased phospho-STAT3 expression (Figure 5A). Consistent with the results of the *in vivo* study, kahweol treatment decreased TGF-β-stimulated phospho-STAT3 expression in AML12 cells and primary hepatocytes, but not in LX2 cells (Figure 5B and 5C).

### Kahweol inhibited phospho-ERK and JNK expression

**Figure 6:**
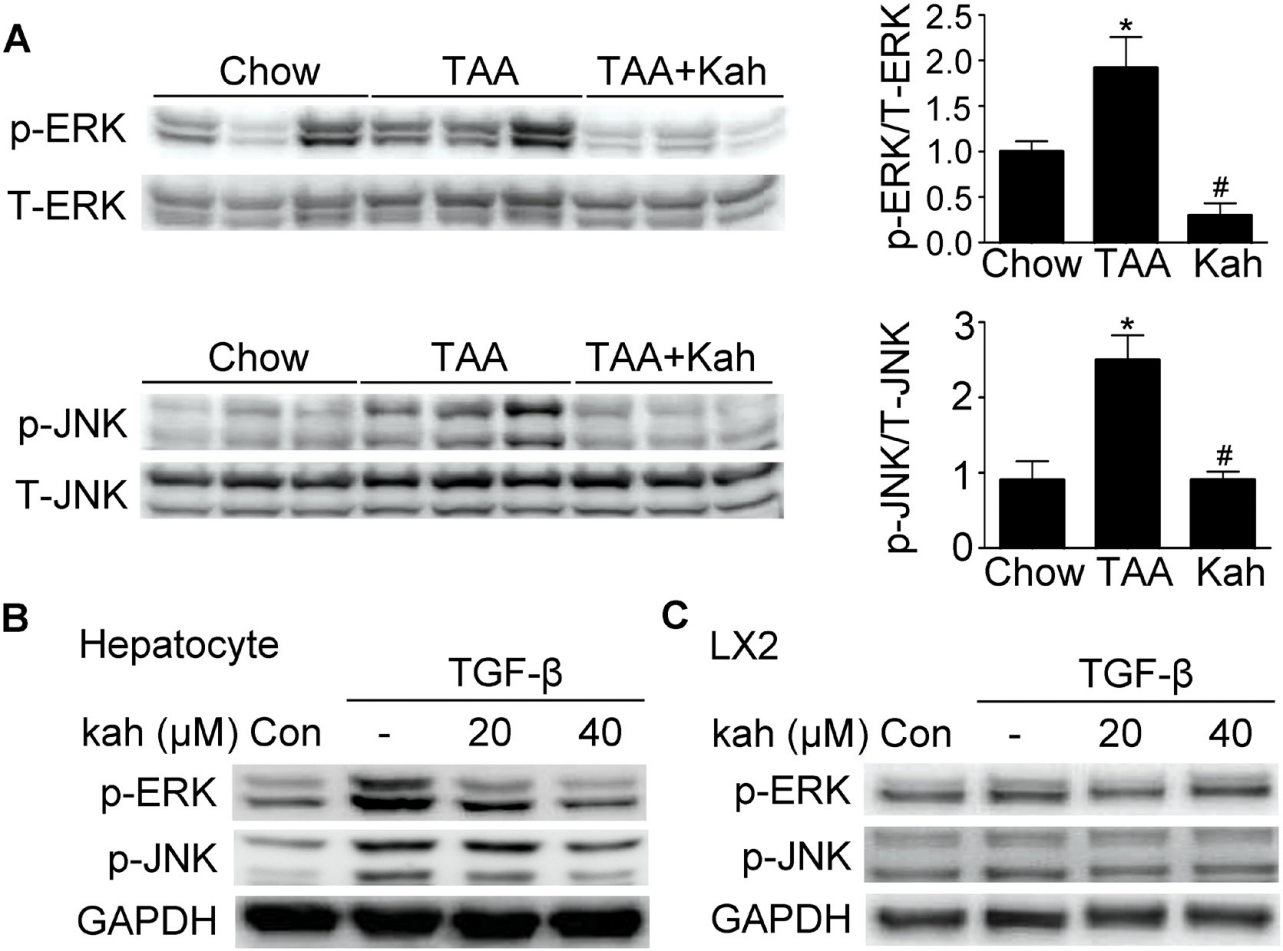
Effect of kahweol on the expression of phospho-ERK and phospho-JNK **(A)** Representative western blot analysis of phospho-ERK and phospho-JNK expression in the liver. Data in the bar graph are mean ± SEM of three independent measurements. ^*^P < 0.05 compared with chow group, ^#^*P* < 0.05 compared with TAA-injection group. **(B)** Western blot analysis showing the effect of kahweol on TGF-β-induced phospho-ERK and phospho-JNK expression. Primary hepatocytes were incubated with various concentrations of kahweol with TGF-β. **(C)** Western blot analysis showing the effect of kahweol on TGF-β-induced phospho-ERK and phospho-JNK expression in LX2 cells

We next examined the effect of kahweol on MAP kinase pathways. Phospho-ERK and JNK expressions were increased in the livers of the TAA-treated group, but decreased in the kahweol-treated group (Figure 6A). Kahweol significantly decreased TGF-β-stimulated phospho-ERK and JNK expression in primary hepatocytes only (Figure 6B and 6C).

## DISCUSSION

The results of this study show that kahweol decreased hepatic fibrosis induced by TAA and inhibited TGF-β-stimulated CTGF expression. The inhibitory effect of kahweol on hepatic fibrosis was associated with downregulation of TGF-β-stimulated phospho-Smad3, STAT3, ERK, and JNK expression.

Coffee consumption is associated with reduced risk and severity of liver disease. Coffee inhibited the progression of liver fibrosis by inhibiting HSC activation and reducing the expression of TGF-β [16, 17, 20–22]. These effects have also been demonstrated in several large-scale human cohort studies. These protective effects of coffee are known to be due to caffeine among the various constituents of coffee. In a study of chronic hepatitis C patients who underwent liver biopsy, consumption of caffeine was associated with lowered risk of liver fibrosis [23]. However, this protective effect was afforded only by caffeine derived from coffee and not that present in the other beverages, including pure caffeine. Therefore, it is expected that coffee contains other constituents besides caffeine that have hepatoprotective effect. Among the various constituents of coffee, kahweol has been considered unhealthy because it was reported to raise the levels of total cholesterol and LDL in a study on a small number of people. However, recent studies have shown that kahweol has various beneficial effects such as anti-cancerous, anti-angiogenic, and anti-inflammatory effects [7, 24–26]. In another study, kahweol decreased hepatotoxicity by decreasing cytochrome P450 2E1 activity in an animal model [27]. In this study, kahweol significantly decreased TAA-induced ECM accumulation *in vivo* and TGF-β-stimulated collagen expression *in vitro*.

CTGF is strongly expressed in fibrotic liver and acts downstream of TGF-β to modulate extracellular matrix production. Earlier studies have shown that coffee decreases CTGF expression and prevents hepatic fibrosis [16, 17]. However, it is unclear what constituents of the coffee have this effect. In this study, kahweol significantly decreased TAA-induced CTGF expression in the liver and TGF-β-stimulated CTGF expression in hepatocytes and HSCs.

Other studies reported that induction of CTGF by TGF-β is mediated via activation of STAT3 and Smad3 [28, 29]. Therefore, we investigated whether the protective effect of kahweol is mediated through the inhibition of TGF- β/Smad3 or TGF-β/STAT3 signaling. In this study, kahweol significantly decreased phospho-Smad3 and STAT3 expressions in the TAA-treated group. Kahweol decreased TGF-β-stimulated phospho-Smad3 and STAT3 expressions in hepatocytes, but not in HSCs. Our results show that hepatocytes also employ the same signaling activity in TGF-β-mediated CTGF expression as HSCs. However, kahweol inhibits this signaling pathway in hepatocytes, but not in HSCs.

CTGF has also been reported to be activated by other signaling molecules such as ERK, JNK, and p38 [30, 31]. In our study, kahweol decreased phospho-ERK and JNK expressions in TAA-induced fibrotic livers and cultured hepatocytes. However, unlike that in hepatocytes, phospho-JNK and ERK expressions did not change after kahweol treatment in LX2 cells. These results suggest that the antifibrotic effect of kahweol in HSCs does not involve these signaling pathways. Therefore, additional studies are needed to elucidate the mechanism of the anti-fibrotic effect of kahweol on HSCs.

In conclusion, we found that kahweol significantly decreased TAA-induced hepatic fibrosis and inhibited TGF-β-stimulated CTGF expression. These results suggest that kahweol may be a new candidate agent for treating liver fibrosis.

## MATERIALS AND METHODS

### Materials

Kahweol was purchased from LKT Laboratories Inc (St Paul, MN, USA). A 40 mM solution of kahweol was initially prepared in DMSO, stored as small aliquots at −20°C, and thawed and diluted in cell culture medium, as required. Thioacetamide (TAA) was purchased from Sigma Aldrich (St. Louis, MO, USA) and recombinant human TGF-β, (5 ng/mL) was purchased from R&D Systems (Minneapolis, MN, USA). The anti-CTGF antibody and anti-phospho-Smad2/3 antibodies were purchased from Santa Cruz (Dallas, TX, USA). Anti-Collagen antibody was purchased from Abcam (Cambridge, UK) and anti-αSMA antibody was purchased from Sigma Aldrich. Anti-GAPDH antibody, anti-phospho-Smad3 (Ser423/425) antibody, anti-phospho-ERK (Th202/Tyr204) antibody, anti-phospho-JNK (Thr182/Tyr185) antibody and anti-phospho-STAT3 (Tyr705) were purchased from Cell Signaling Technology (Beverly, MA, USA).

### Animal study

Male 8-week-old C57BL/6 mice were purchased from Hanasangsa (Pusan, Korea) and housed in a facility equipped with 12 h light/dark cycle. All procedures were performed in accordance with the institutional guidelines for animal research (KM-2013-51R1). Kahweol was administered orally premixed with the milled pellet (FEEDLAB, Guri, Korea). Mice were divided into three groups and were fed either a chow diet (control, n=5), a chow diet with TAA injection (TAA 200 mg/kg of body weight, n=7), or a chow diet supplemented with kahweol (75 mg/kg) and TAA injection (TAA+Kah, n=7). Liver fibrosis was induced by intraperitoneal injection of TAA dissolved in saline, thrice a week for 8 weeks. Kahweol was administered orally, premixed with the milled pellet (FEEDLAB, Guri, Korea). We measured body weight and food intake of mice three times a week. After 8 weeks, animals were euthanized forthe collection of blood and liver tissue samples.

### Cell culture

Mouse hepatocyte AML-12 cell line was purchased form the American Type Culture Collection (Manassas, VA). AML12 cells were cultured in 5% CO_2_/95% air at 37°C in DMEM/F12 medium (GIBCO-BRL, Grand island, NY) supplemented with 10% fetal bovine serum (Hyclone, Logan, UT), insulin-transferrin-selenium (ITS; GIBCO-BRL), dexamethaosone (40 ng/mL; Sigma) and antibiotics (Anti-Anti: GIBCO-BRL). LX2, human hepatic stellate cell line, was a kind gift from Dr. Jeong (Korea Advanced Institute of Science and Technology, Daejeon, Korea). The LX2 cells were cultured in 5% CO_2_/95% air at 37°C in DMEM (GIBCO-BRL) supplemented with 10% fetal bovine serum and antibiotics. Cells were treated with chemicals in 0.5% FBS with or without TGF-β (5 ng/ml), and then subsequently processed for isolation of protein and RNA as described below.

### Primary cultures of hepatocytes

C57BL/6 hepatocytes were isolated by perfusing the liver via the portal vein. The liver was perfused with resuspension buffer (5.4 mmol/L KCl, 0.44 mmol/L KH_2_PO_4_, 140 mmol/L NaCl, 0.34 mmol/L Na2HPO_4_, 0.5 mmol/L EGTA, 25 mmol/L Tricine, pH 7.2) at 5 mL/min for 10 min and then perfused with collagenase solution [collagenase type I (Worthington Biochemical Corp, Freehold, New Jersey), 0.75mg/ml, pH 7.2] at 5 mL/min for 10 minutes. After perfusion, the liver was shaken for 20 min at 37°C and filtered through a mesh (85 μm nylon mesh). Hepatocytes were collected by centrifugation at 42 ×*g* for 5 minutes at 4°C, resuspended in William's E medium (Sigma), and seeded onto type I collagen-coated 60-mm dishes (IWAKI Scitech Kiv, Tokyo, Japan) at a density of 5 × 10^5^ cells/mL. The viability of hepatocytes, as measured by trypan blue dye exclusion, was always greater than 85%. After a 2- to 3-hour incubation, the medium was exchanged with DMEM. Hepatocytes were treated with chemicals in 0.5% FBS with or without TGF-β (5 ng/ml), and then subsequently processed for isolation of protein and RNA as described below.

### Histological and immunohistohistochemical analysis

Liver tissue was fixed in 4% formaldehyde solution and embedded in paraffin, and 4-μm section were cut. Liver sections were deparaffinized in xylene and rehydrated through graded ethanol concentration.

Histochemical staining was performed with H&E and Sirius Red. For Sirius red staining. Immunohistochemical staining was performed using anti-Collagen, anti-αSMA, anti-phospho-Smad2/3 and anti-CTGF primary antibodies followed by horseradish peroxidase conjugated anti-mouse, anti-rabbit, or anti-goat IgG secondary antibodies (Dako, Glostrup, Denmark), according to the manufacturer's instructions. All data were normalized to the chow diet (=1) and in all bar graphs were expressed as fold increase relative to the chow diet.

### Quantitative real-time (qRT)-RCR

Total RNA was isolated from cells and tissue extracts, using the Trizol reagent (Invitrogen, MA, USA). Reverse transcription was performed using the Maxima First Strand cDNA synthesis kit (Thermo scientific, MA, USA). Quantitative real-time RT-PCR was performed using a SYBR Green PCR master mix kit (Roche Diagnostics, Indianapolis, Indiana) and a Light Cycler 96 instrument (Roche Diagnostics). PCR parameters were as follows: 45 cycles of 95°C for 30 s, 60°C for 10 s, and 72°C for 15 s. Primer sequences were as follows: human Collagen forward, 5′-CACACGTCTCGGTCATGGTA-3′, and reverse, 5′-AAGAGGAAGGCCAAGTCGAG-3′; mouse collagen forward, 5′- GCCTTGGAGGAAACTTTGCTT- 3′ and reverse, 5′- GCACGGAAACTCCAGCTGAT -3′; human CTGF forward, 5′-CACCCGGGTTACCAATGACA-3′, and reverse, 5′-TCCGGGACAGTTGTAATGGC-3′; mouse CTGF forward, 5′-CCAGACCCAACTATGATGCG-3′, and reverse, 5′-GTGTCCGGATGCACTTTTTG-3′; mouse TGF-β forward, 5′-AAATCAACGGGATCAGCCCC-3′, and reverse, 5′-GGATCCACTTCCAACCCAGG-3′; mouse αSMA forward, 5′ – CAGGCTGTGCTGTCCCTCTA - 3′, and reverse, 5′- CGGCAGTAGTCACGAAGGAA - 3′; human GAPDH forward, 5′-GGAGCCAAAAGGGTCATCAT-3′, and reverse, 5′-GTGATGGCATGGACTGTGGT-3′; mouse GAPDH forward, 5′- ACGACCCCTTCATTGACCTC -3′, and reverse, 5′- ATGATGACCCTTTTGGCTCC-3′. GAPDH was used as an internal standard.

### Western blot analysis

Cells were harvested in lysis buffer [50 mM Tri-HCl (pH 8.0), 150 mM NaCl, 1 mM EDTA, 1% TritonX-100, and 0.5% Na-deoxycholate] containing protease/phosphatase inhibitors and dithiothreitol. The cells were lysed on ice for 30 min and lysate was collected by centrifugation at 13,000 rpm for 10min. Protein quantification was performed using a BCA protein assays (Thermo scientific). Cell lysates of 10 μg were separated by SDS-PAGE and then transferred electrophoretically to a polyvinylidene difluoride membrane (Millipore, Bedford, MA). The membrane was incubated serially in a blocking buffer (5% skim milk in Tris-buffered salin contain 0.1% Tween 20), primary antibody and appropriate horseradish peroxidase-conjugated secondary antibody, and then developed using the Clarity^™^ Western ECL substrate kit (Bio-Rad, Richmond, CA). The membrane was re-probed with anti-GAPDH to verify equal loading of protein in each lane. Signal intensities were quantitated by densitometry using the ImageJ software (NIH, Bethesda, MD, USA).

### Serum biochemical assays

The serum AST and ALT levels were measured using an auto-chemical analyzer (Hitachi 747, Tokyo, Japan) and an ultraviolet assay method.

### Statistical analysis

Data are expressed as means ± SEM. ANOVA was used to determine significant differences in multiple comparisons and was performed by the Duncan test. Values of P <0.05 were considered statistically significant

## SUPPLEMENTARY FIGURE


