# Supplementary material for: Kahweol decreases hepatic fibrosis by inhibiting the expression of connective tissue growth factor via the transforming growth factor-beta signaling pathway
Source: Oncotarget. 2017 Aug 1;8(50):87086–94. doi: 10.18632/oncotarget.19756 (PMC5675617; doi:10.18632/oncotarget.19756)
Supplement: Supplementary file 1 [file oncotarget-08-87086-s001.pdf]

# Kahweol decreases hepatic fibrosis by inhibiting the expression of connective tissue growth factor via the transforming growth factor-beta signaling pathway

## SUPPLEMENTARY MATERIALS

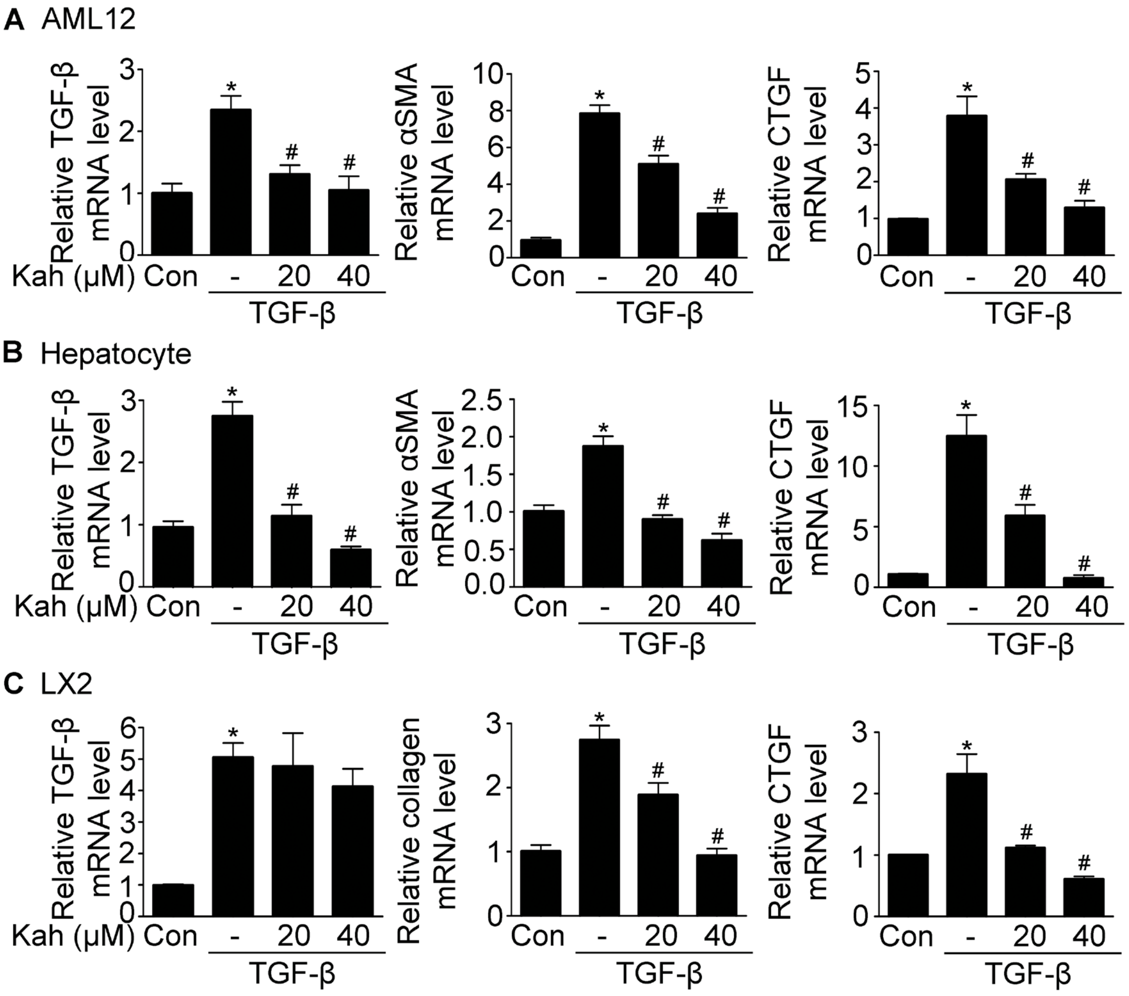


**Supplementary Figure 1: Effect of kahweol on TGF-β-stimulated TGF-β, α-SMA, collagen and CTGF mRNA expression. (A)** Representative real-time RT-PCR analysis of TGF-β, α-SMA and CTGF mRNA expression in AML12 cells. Data in the bar graph are mean ± SEM of three independent measurements. $^{*}P < 0.05$ compared with control, $^{\#}P < 0.05$ compared with TGF-β only. **(B)** Representative real-time RT-PCR analysis of TGF-β, α-SMA and CTGF mRNA expression in primary hepatocyte cells. Data in the bar graph are mean ± SEM of three independent measurements. $^{*}P < 0.05$ compared with control, $^{\#}P < 0.05$ compared with TGF-β only. **(C)** Representative real-time RT-PCR analysis of TGF-β, type I collagen and CTGF mRNA expression in LX2 cells. Data in the bar graph are mean ± SEM of three independent measurements. $^{*}P < 0.05$ compared with control, $^{\#}P < 0.05$ compared with TGF-β only
